# Oxygen-dependent regulation of tumor growth and metastasis in human breast cancer xenografts

**DOI:** 10.1371/journal.pone.0183254

**Published:** 2017-08-23

**Authors:** Kristine Yttersian Sletta, Maria K. Tveitarås, Ning Lu, Agnete S. T. Engelsen, Rolf K. Reed, Annette Garmann-Johnsen, Linda Stuhr

**Affiliations:** 1 Department of Biomedicine, University of Bergen, Bergen, Norway; 2 Center for Cancer Biomarkers (CCBIO), University of Bergen, Bergen, Norway; 3 Matrix Biology, Haukeland University Hospital, Bergen, Norway; University of South Alabama Mitchell Cancer Institute, UNITED STATES

## Abstract

**Background:**

Tumor hypoxia is relevant for tumor growth, metabolism, resistance to chemotherapy and metastasis. We have previously shown that hyperoxia, using hyperbaric oxygen treatment (HBOT), attenuates tumor growth and shifts the phenotype from mesenchymal to epithelial (MET) in the DMBA-induced mammary tumor model. This study describes the effect of HBOT on tumor growth, angiogenesis, chemotherapy efficacy and metastasis in a triple negative MDA-MB-231 breast cancer model, and evaluates tumor growth using a triple positive BT-474 breast cancer model.

**Materials and methods:**

5 x 10^5^ cancer cells were injected s.c. in the groin area of NOD/SCID female mice. The BT-474 group was supplied with Progesterone and Estradiol pellets 2-days prior to tumor cell injection. Mice were divided into controls (1 bar, pO_2_ = 0.2 bar) or HBOT (2.5 bar, pO_2_ = 2.5 bar, 90 min, every third day until termination of the experiments). Treatment effects were determined by assessment of tumor growth, proliferation (Ki67-staining), angiogenesis (CD31-staining), metastasis (immunostaining), EMT markers (western blot), stromal components collagen type I, Itgb1 and FSP1 (immunostaining) and chemotherapeutic efficacy (5FU).

**Results:**

HBOT significantly suppressed tumor growth in both the triple positive and negative tumors, and both MDA-MB-231 and BT-474 showed a decrease in proliferation after HBOT. No differences were found in angiogenesis or 5FU efficacy between HBOT and controls. Nevertheless, HBOT significantly reduced both numbers and total area of the metastastatic lesions, as well as reduced expression of N-cadherin, Axl and collagen type I measured in the MDA-MB-231 model. No change in stromal Itgb1 and FSP1 was found in either tumor model.

**Conclusion:**

Despite the fact that behavior and prognosis of the triple positive and negative subtypes of cancer are different, the HBOT had a similar suppressive effect on tumor growth, indicating that they share a common oxygen dependent anti-tumor mechanism. Furthermore, HBOT significantly reduced the number and area of metastatic lesions in the triple negative model as well as a significant reduction in the EMT markers N-cadherin, Axl and density of collagen type I.

## Background

Breast cancer is the leading cause of cancer death among women worldwide and 90% of breast cancer deaths are due to metastasis [1]. The prognosis of breast cancer is classified by variables such as histological type, grade, stages and receptor status. The receptor status can be classified as estrogen receptor-α (ER), progesterone (PR) and human epidermal growth factor 2 (HER2) positive (also called triple positive or Luminal B).The tumors that do not express the receptors (ER, PR or HER2) are also called triple negative tumors, while the HER2 positive and Luminal A with ER, PR positive and HER2 negative consitute separate subgroups. The molecular subtypes of breast cancer have been shown to predict the clinical outcome, and in particular breast cancers expressing hormone receptors (luminal A) have a more favorable prognosis, while women with basal-like breast cancers (including most of the triple-negative tumors) had shorter relapse-free survival times than women with other types of breast cancer [2]. Despite differences in taxonomy, there is a consistent trend across all studies confirming the relatively poor prognosis of the triple-negative or basal-like breast cancer subgroup, and patients with triple-negative breast cancer, carries the worst prognosis among all subtypes due to the highly aggressive and metastatic potential of this subtype [3]. As such, many preclinical and clinical studies have focused on developing effective therapeutics for this group of patients.

The main features of metastasis is that primary tumor cells migrate to and invade distant sites through multiple steps. Accumulating evidence has shown that the hypoxic tumor microenvironment plays a key role in regulating breast cancer progression and metastasis, in particular via hypoxia-inducible factor 1 (HIF-1), a master regulator of the hypoxic response [4]. Enhanced oxygen tension will degrade HIF-1 and thereby shut off genes that are generally stimulated by the hypoxic tumor microenvironment [5]. In addition, hypoxia is considered to be important for initiating the epithelial-mesenchymal transition (EMT), where cancer cells lose key epithelial markers such as E-cadherin and obtain mesenchymal markers including N-cadherin. Tumor cells undergoing EMT change morphology from a cobble-stone like phenotype to a flat and spindle-shaped mesenchymal type with high level of motility proteins, enabling invasion, extravasation and metastatic niche formation [6]. Thus, there is a close relationship between hypoxia and tumor metastasis.

Hypoxia is considered critical for tumor progression. Our group has previously shown that the opposite, hyperoxia (HBOT-hyperbaric oxygen treatment) attenuates or significantly reduces breast cancer growth using a chemically induced (DMBA) (triple positive) model and a murine triple negative (4T1) adenocarcinoma model [7–11]. Furthermore, our studies demonstrated that hyperoxia induced a mesenchymal-to-epithelial transition (MET) in the DMBA breast cancer model [12]. These findings, together with a significantly decreased collagen deposition in the tumors, indicated that HBOT induced a less aggressive tumor behavior [12]. In addition, a study showed a reduction in tumor interstitial pressure with a concomitant increase in uptake of the chemotherapeutic agent 5FU after HBOT [8].

Thus, the present study aimed to investigate the effect of HBOT on tumor growth of two different receptor subtypes of human breast tumors; the triple positive BT-474 and the triple negative MDA-MB-231 model. In addition, the effect of HBOT on 5FU efficacy, as well as the development of metastatic lesions was investigated in the latter model.

## Materials and methods

### The cell-lines and culture

The human breast cancer cell lines, MDA-MB-231 and BT-474 were obtained from American Type Culture Collection (Rockville, MD, USA) and grown in F12K (Bio-Whittaker, Verviers, Belgium) or RPMI medium, respectively, supplemented with 100 ml/L of fetal calf serum, 100 U/L of penicillin and 100 mg/L of streptomycin (Sigma-Aldrich, Steinheim, Germany). The cells were amplified as a monolayer in plastic tissue culture flasks 75 cm^2^ (NUNC, Roskilde, Denmark) in a humidified incubator set at 37°C with 5% CO_2_ and 95% air, and were seeded until approximately 80% confluence.

### Animals

Female NOD/SCID mice (Jackson Laboratory, USA), age 7–10 weeks, weighing 20–25 g were used. The mice were kept in individually ventilated, pathogen-free cages where they had free access to food and water *ad libitum*. All the experiments were approved by the Norwegian Committee for Animal Research (FOTS project number: 23135440). Bodyweight, activity, tumor size and skin changes were monitored carfully throughout the experiments. No animals showed any signs of illness following tumor formation and metastasis and none died due to the experimental procedure.

### Mouse model for human breast cancer xenografts

For the xenograft experiments, 5 x 10^5^ tumor cells, in 0.15 ml PBS were injected subcutaneously on one side and into the fat pad of their mammary crest in the groin area. For the BT-474 xenograft, sixty-day release pellets containing 17β-Estradiol (1.7 mg) and progesterone (10 mg) (Innovative Research of America, Sarasota, FL) were implanted subcutaneously in the neck 2 days prior to injecting the cells. By day 8, when 100% of the mice had developed palpable tumors, they were assigned to either hyperbaric oxygen treatment (HBOT) or control.

Tumor volume was measured every three days using a caliper. The location of the tumor only allowed measurements in two dimensions. The best estimate of tumor volume given these restrictions was a calculation based on a cylindrically shaped tumor according to the following equation: Tumor volume (mm^3^) = (short axis)^2^ x (long axis) x (π/6).

After completion of the study, the mice were sacrificed with CO_2_ when fully anesthetized using Ketalar-Dormicum and the whole tumor was excised and processed for subsequent immunohistochemistry and western blot analysis.

### Hyperbaric oxygen treatment (HBOT)

A hyperbaric animal research chamber (27 liter, OXYCOM 250 ARC, HYPCOMOY, Tampere, Finland) was used. The chamber was flushed with pure O_2_ for approximately 15 minutes at normal ambient pressure. The pressure was then slowly increased from 1 to 2.5 bar (equivalent to 15 msw) during approximately 10 minutes, and was kept stable at this pressure for another 90 minutes. To ensure levels above 97%_,_ the chamber was flushed with pure O_2_ every 10 minutes for 5 min. After the treatment the chamber was slowly decompressed to 1 bar over a period of approximately 15 minutes. The mice underwent this treatment on days 1, 4, 7, 10, 13 and 16. Day 1 (starting measurements) was set as being 8 days after the injection of tumor cells. For the separate metastatic study, the mice underwent 17 treatments, *i*.*e*. every third day, during the whole 54 day period.

### 5-Fluorouracil (5FU)

To evaluate if HBOT could potentiate drug efficacy, the chemotherapeutic drug Fluorouracil, 5FU (50 mg/ml, Hospira Nordic AB, Stockholm, Sweden) was administered *i*.*v*. at a dose of 1.5 mg/kg in a volume of 0.1 ml as a stand-alone treatment or in combination with HBOT. In the latter case it was given immediately prior to each HBOT for optimal effect.

### CD31-staining

On the last day of measurements, the tumors were quickly dissected out, snap-frozen in liquid nitrogen and stored at –80°C until further use. The frozen tumor tissue was embedded in Tissue Tek (Sakura Fintek Europe, Zoeterwounde, the Netherlands) and cut into 10–20 μm slices with a cryostat microtome (Leica CM 3050 S-Cryostat, Nussloch, Germany). Blood vessels in the frozen slides of tumor (10 μm) were immunostained with rat anti-mouse CD31 (1:200) (AbD serotec, Morphosys UK Ltd, Oxford, UK) as primary antibody. Biotinylated rabbit-anti-rat (Vectastatin ABC kit, peroxidase Rat IgG PK 4004, Bioteam AS, Trondheim, Norway) was used as secondary antibody. Rabbit serum was used as a blocking agent and H_2_O_2_ in methanol was used to quench endogenous peroxidases. An avidin biotin peroxidase complex was used to bind to the biotinylated secondary antibody. The chromogen DAB was used to visualize the blood vessels (DAB, Sigma-Aldrich, Germany). Richardsson’s stain was used to counterstain the rest of the tumor tissue. The cross-section of CD31 positive structures was quantified per mm^2^ of the entire tumor. The blood vessel diameter was also measured. The Computer-software program NIS-Elements AR 3.2 64-bit (Laboratory Imaging Ltd, Prague, Czech Republic) was used for this purpose. Also, for standard histologic examination of the tumor specimens, frozen sections from both HBOT tumors and controls were stained with Haematoxylin-Eosin.

### Ki67-staining

Ki67-staining was carried out on 10 μm thick cryosections from the control group and the HBOT group. Sections were fixed for 8 minutes in 70% methanol and 30% acetone at -20°C, and further washed 3 x 10 min in PBS. Unspecific binding was blocked with Peroxidase Block from the Dako EnVision+ System-HRP (DAB+) kit (K4006, Dako, Glostrup, Denmark) for 8 minutes, before washing the sections 2 min x 3 in PBS. The primary antibody, Monoclonal Mouse Anti-Human Ki67 (M7240, Dako, Glostrup, Denmark), was diluted 1:75 in Antibody Diluent with Background Reducing Components (S3022, Dako, Glostrup, Denmark), and the sections were incubated 1 hr at RT. Thereafter, the sections were washed 5 min x 2 with PBS-T, and 5 min x 1 with PBS. The Peroxidase Labeled Polymer from the Dako EnVision+ System-HRP (DAB+) kit (K4006, Dako, Glostrup, Denmark) was applied and incubated for 30 min at RT, before washing 5 min x 2 with PBS-T. Liquid DAB+ Chromogen and buffer from the Dako EnVision+ System-HRP (DAB+) kit (K4006, Dako, Glostrup, Denmark) was mixed as described by manufacturer, and incubated on sections for 1 min and 50 sec. Sections were immediately washed in ddH_2_O for 3 min. Hematoxylin was used as a counterstain, with 3 min incubation prior to 3 min wash in 37°C running water. Sections were dehydrated for 10 sec in each bath in the following order; 75% ethanol, 96% ethanol, 96% ethanol, 100% ethanol, 100% ethanol, Xylol, Xylol. Finally, the sections were mounted with coverslips using Hecht Karl^TM^ Assistent Histokitt (Fisher Scientific, Göteborg, Sweden). Images were captured and analysed using the Nikon Eclipse E600 microscope, and the computer program NIS-Elements AR 3.2 64-bit (Laboratory Imaging Ltd, Prague, Czech Republic). Ki67 positive cells were counted as positive cells per mm^2^.

### Immunofluorescence (IF) staining of tissue sections

IF staining was performed on cryosections of tissue samples from mice treated with or without HBO for 24 days (5 mice from each group). Sections with a thickness of 10 μm were fixed in methanol for 10 minutes at -20°C and then rehydrated in PBS (3x10 min). Unspecific binding sites were blocked with 10% goat serum diluted in PBS for 1 h at room temperature. Thereafter, the sections were incubated with primary antibody against integrin β1, rat anti-mouse (Millipore MAB1997, dilution 1:400), rabbit anti-FSP1 antibody (Millipore 07–2274, dilution 1:400), rabbit anti-mouse collagen type I (Millipore AB765P, dilution 1:200), rabbit anti-human cytokeratin-7 (Novus Biologicals NBP1-30152, dilution 1:400) or mouse anti-human cytokeratin (clones AE1/AE3, Dako M3515, dilution 1:400) for 1 h at 37°C. After two washing steps with PBS/0.05% Tween and an additional washing step with PBS, the secondary antibodies were applied. Alexa Fluor® 488 Goat Anti-Rat (Jackson ImmunoResearch Cat.112-545-003, dilution 1:800) was used for integrin β1, Alexa Fluor® 594 Goat Anti-Rabbit (Jackson ImmunoResearch Cat.111-585-144, dilution 1:800) was used for collagen type I or cytokeratin-7. The sections were incubated with the secondary antibody for 1 h at room temperature. Sections stained with secondary antibody only were used as negative controls. After two washing steps in PBS/0.05% Tween, sections were mounted in ProLong® Gold Antifade Mountant with DAPI (ThermoFisher P36935) and were dried overnight. Afterwards, the mounted sections were observed under a Zeiss Axioscope fluorescence microscope and photographed using a digital AxioCam mRM camera (Zeiss). Four to five sections from each tumor was used for analysis using the software program Image J (National Institite of Health, MD, USA).

For IF staining to evaluate Axl RTK and N-cadherin, cryosections were air dried 40 min at room temperature (RT), and subsequently fixed in 3.7% formalin for 10 min, RT. Sections were washed 2 x 5 min with PBS, and incubated with 0.5% Triton X-100 in PBS, 4 min, RT. Following 2 x 5 min washes, sections were blocked with 5% goat serum, 1% BSA and 0.1% Triton X-100 in PBS for 1 h, RT. Sections were incubated with primary antibodies over night at 4°C; mouse anti human Axl (1H12, BerGenBio AS, dilution 1:150), rabbit anti human N-cadherin (Abcam, ab18203, 1:200). Following wash 3 x 10 min with 0.1% Triton X-100 in PBS, sections were incubated with secondary antibodies for 1 h at RT; goat anti mouse-488 (A21131, Invitrogen/Molecular probes, dilution 1:200) and goat anti rabbit 647 (A21244, Invitrogen/Molecular probes, dilution 1:300). Sections were washed with Triton X-100 in PBS 3 x 10 min, dipped in water and mounted with ProLong Diamond antifade mounting medium with DAPI (P36962, Life technologies/Molecular Probes) and high precicion cover glass (No. 1014, Assistent). Images were obtained on a Leica TCS SP5 confocal microscope system, equipped with a 63x HCX PL Apo Oil objective (Leica). A maximum projection snapshot of a 9 μm z-stack is shown. The confocal microscope is available through the molecular imaging centre (MIC), a core facility at the University of Bergen.

### Metastasis

Development of metastasic lesions was studied in a separate series of experiements. Mice were injected with 5 x 10^5^ MDA-MB-231 cells and terminated at day 54 post-injection. The lungs were fixed post mortem using approximately 1 mL of Bouin’s solution (Gurr BDH Chemicals Ltd., Poole, UK) injected into the trachea. The lungs were immediately dissected out, fixated in new Bouin’s solution, washed in 70% ethanol, dehydrated and embedded in paraffin using standard procedures. The liver and femur bone were removed and fixed in formalin immediately after sacrificing the animal. After fixation the bone was decalcified in 10% EDTA, pH 7.2, during a period of 5 weeks. Sections were stained with H & E staining and examined by light microscopy. In order to quantify lung metastasis, 4 coronal sections from both lungs from each animal were examined. Total number of metastasis per lung was counted, and the area per lung covered by metastasis was measured in mm^2^ (Nikon Digital Sight, Nikon Corporation).

### Western blot

On the last day of measurements the tumors were dissected out, snap frozen in liquid nitrogen and stored at -80°C until further use. The MDA-MB-231 tumors were divided in two halves, and one was used for immunohistochemistry and the other to make the proteinlysate for western blot. Briefly, a cross section (approximately 50 mg) of tumor tissue was homogenized in denaturing lysis buffer (DLB), containing 50 mM Tris-HCl (pH 7.4), 150 mM NaCl, 1% Trition X, complete EDTA free tablet and phosphatase inhibitor, with homogenizing beads in a tissue homogenizer (Precally® 24, Bertin Technologies, France) for 6,800-3x10-30 at 4°C and incubated on ice for 45 min. The lysate was removed and centrifuged at 12,000 rpm for 10 min at 4°C (Eppendorf 5415R, Hamburg, Germany). The supernatant was collected, aliquoted and stored at -80°C. The protein concentration was determined by a BCA assay (Pierce^TM^, Thermo Scientific, Rockford, USA), according to the manufacturer’s protocol. Equivalent concentrations were run on a 12% gel under reducing conditions with 50 mM DTT in sample buffer, and transferred using iBlot^TM^ (Invitrogen, Life Technologies, Carlsberg, CA, USA) for 10 min. Membranes were blocked with I-block (Tropix®, Thermo Scientific, Bedford, USA) for 1 h and 30 min at RT. Rabbit anti-mouse E-cadherin (ab 53033, diluting factor 1:800) and rabbit anti-mouse N-cadherin (ab76057, diluting factor 1:1000) (Abcam, Cambridge, UK) were used as primary antibodies and incubated over night at 4^°^C. Membranes were washed further for 3 x 5 min in TBS-T and incubated with secondary antibody (goat anti-rabbit IgG HRP, ab97051, diluting factor 1:5000)(Abcam, Cambridge, UK) for 2 h at RT. Membranes were washed 2 x 5 min TBS-T and 1 x 5 min TBS before development with ECL substrate (Pierce ECL Western Blotting Substrate, Thermo Scientific, 32209) chemiluminescense was measured with Molecular Imager ChemiDoc XRS+ (BIO-RAD). The ImageLab v5.2.1 (BIO-RAD) software was used for analysis and quantification of the results.

### Statistical analysis

Sigmaplot 12.5 (Systat Software inc) was used for statistical analysis. Either the unpaired two-tailed t-test, or the Mann-Whitney rank sum test, was used to analyze statistical differences between the two groups. One-way Anova for 5FU experiemenst followed by posthoc test was used. Results were accepted as statistically significant when p < 0.05.

## Results

### Growth of different molecular subtypes of breast cancer during HBOT

**Fig 1:**
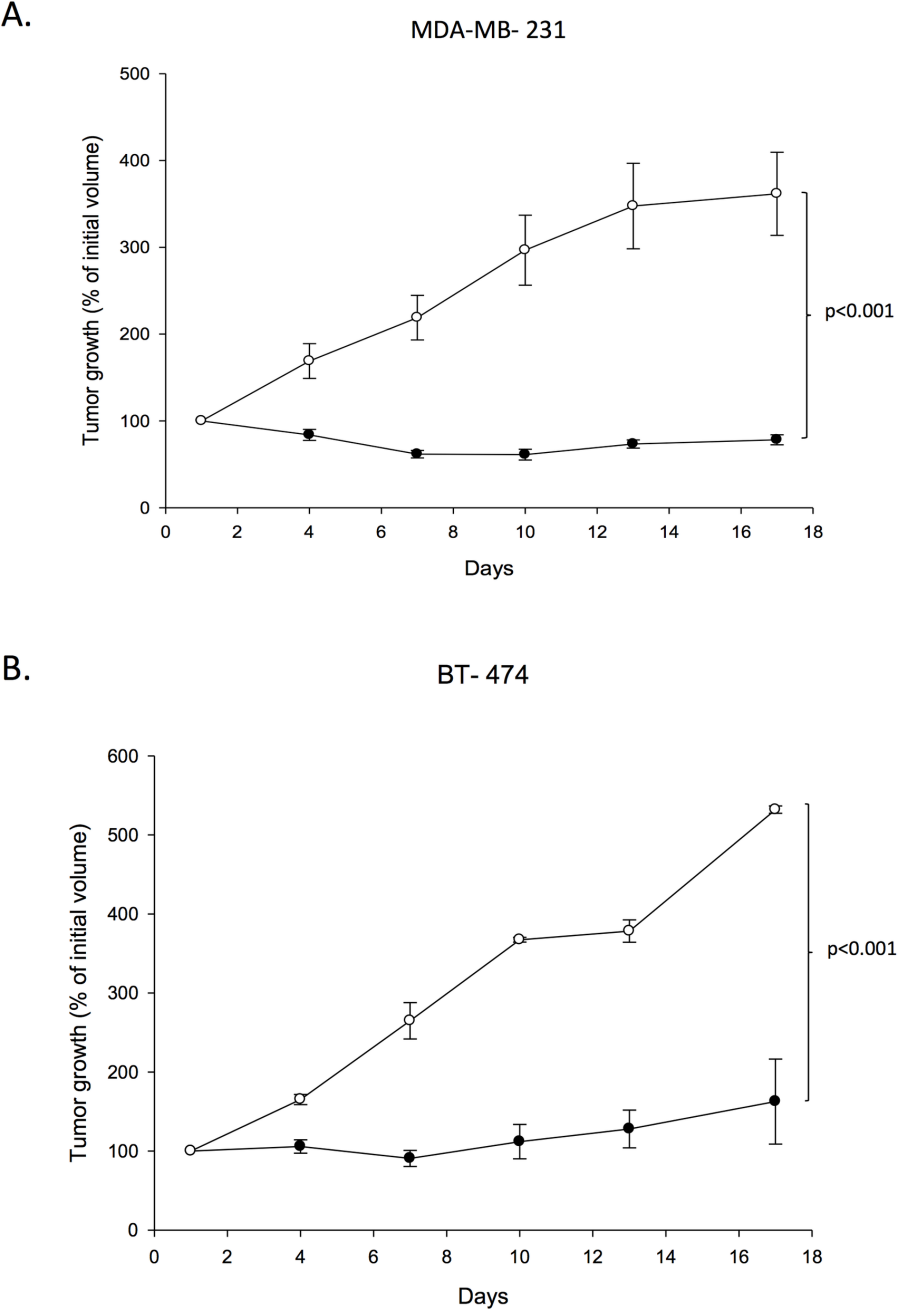
Tumor growth. The effect of hyperbaric oxygen treatment on the growth of triple negative MDA-MB-231 (p<0.001) (A) and triple positive BT-474 (p<0.001) (B) tumors. Cells were injected subcutaneously in the mammary fat pad. Mice were treated with 2.5 bar pure oxygen, 90 min each time, every third day, during a period of 24 days (measurements started day 7 post injection) (closed circles) and compared to controls (open circles). Mean ± SEM. n = 7 in each group.

We assessed the anti-tumorigenic activity of HBOT compared to control on mouse xenograft experiments using the triple negative MDA-MB-231 (n = 14) and triple positive BT-474 (n = 14) human breast cancer cell lines. The mice were exposed to either normal atmospheric pressure or given HBOT, and the tumor volume was measured every 3 days for 17 days. HBOT significantly (p<0.001) suppressed the tumor growth of both the triple-negative MDA-MB-231 and triple positive BT-474 tumors during a treatment period of 17 days (24 days post injection) as indicated in Fig 1, panels A-B. The results demonstrated the inhibitory effect of HBOT on tumor growth to be independent of breast cancer subtypes indicating a shared oxygen dependent antitumor mechanism. The size of the MDA-MB-231 tumors were 692 ± 106 mm^2^ and 461 ± 55 mm^2^ in controls and HBOT mice respectively at day 53 (final day) in the separate metastatic study.

### HBOT significantly reduced the number of proliferating cells

**Fig 2:**
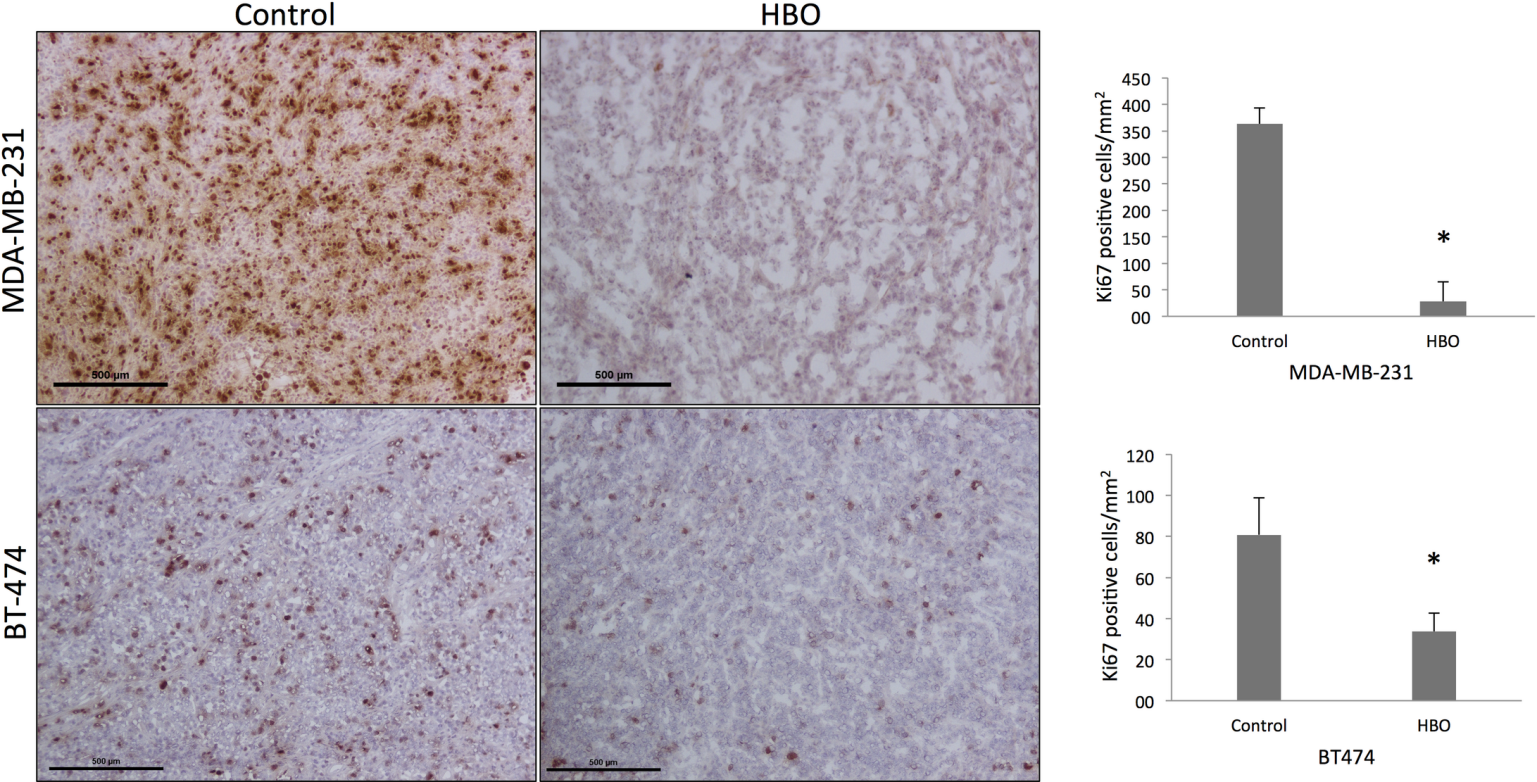
Proliferation. Ki67-staining revealed a significantly higher number of proliferating cells in the control group compared to the HBOT group in both models (MDA-MB-231, * p = 0.0002. BT-474, * p = 0.0001). Scale bar 500 μm.

Ki67-staining, presented in Fig 2, revealed a significantly higher number of proliferating cells in the control group compared to the HBOT group, with an average of 363 (n = 4) and 28 (n = 3) positive cells per mm^2^, respectively (p = 0.0002) in the MDA-MB-231 group and an average of 80.8 (n = 4) and 33.6 (n = 4) positive cells per mm^2^, respectively (p = 0.0001) in the BT-474 group.

### HBOT did not affect tumor blood vessel density or diameter

Since angiogenesis is known to greatly influence tumor growth, blood vessels were stained using rat anti-mouse CD31 as primary antibody. The average tumor blood vessel density and blood vessel diameter are given in Table 1. There was no statistically significant difference between the HBOT tumors and controls. Based on this, we conclude that the tumor growth suppression during HBOT is not attributed to any effect on angiogenesis in these human tumor xenograft models.

**Table 1:** Blood vessels.

	MDA-MB-231		BT-474	
	Controls	HBO	Controls	HBO
**Pressure pO**_**2**_	**1 bar**	**2.5 bar**	**1 bar**	**2.5 bar**
**% O**_**2**_	**20**	**100**	**20**	**100**
Blood vessels density (number/mm^2^)	13.5 ± 1.6	12.4 ± 1.8	11.1 ± 2.2	13.1 ± 4.5
Blood vessel diameter (μm)	7.3 ± 1.7	7.2 ± 1.8	12.0 ± 6.1	12.3 ± 6.4

Blood vessel density and diameter in MDA-MB-231 and BT-474 tumors in control and after hyperbaric oxygen treatment for 24 days. Means ± SD.

### HBOT does not enhance the 5FU efficacy

**Fig 3:**
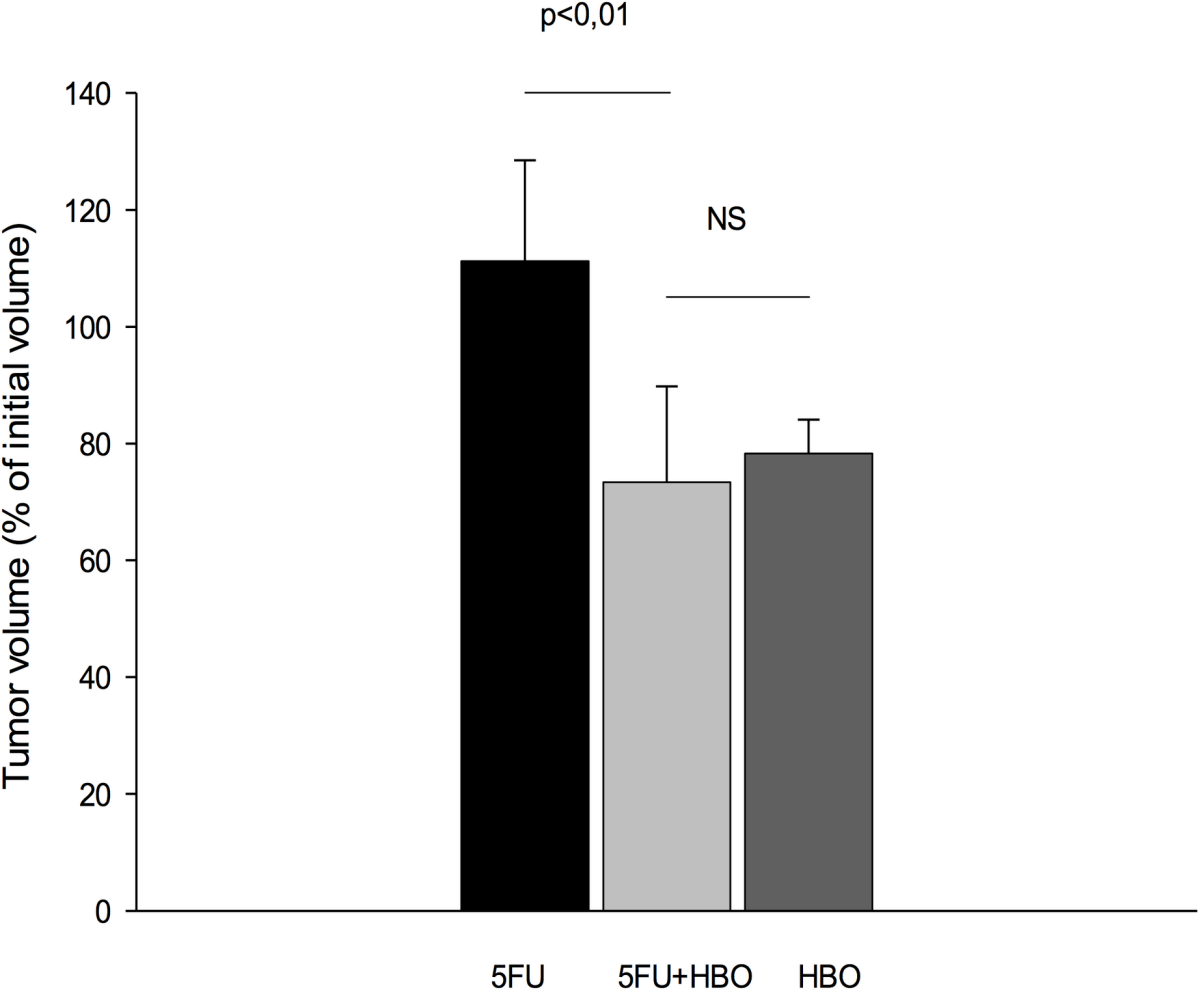
5-Fluorouracil. The effect of hyperbaric oxygen treatment (HBOT) (n = 10), the chemotherapeutic drug 5FU (1.5 mg/kg in 0.1 ml) (n = 10) or 5FU in combination with HBOT (n = 10) on growth of MDA-MB-231 human mammary tumors over a period of 17 days. *p<0.05.

Tumor hypoxia is known to be connected to drug resistance and thus we investigated if hyperoxia might influence the efficacy of the chemotherapeutic drug 5FU. As a stand-alone treatment, 5FU significantly reduced tumor growth in the MDA-MB-231 model as expected. Nevertheless, HBOT did not potentiate the effect of 5FU in the present MDA-MB-231 model (Fig 3).

### HBOT affects metastasis

**Fig 4:**
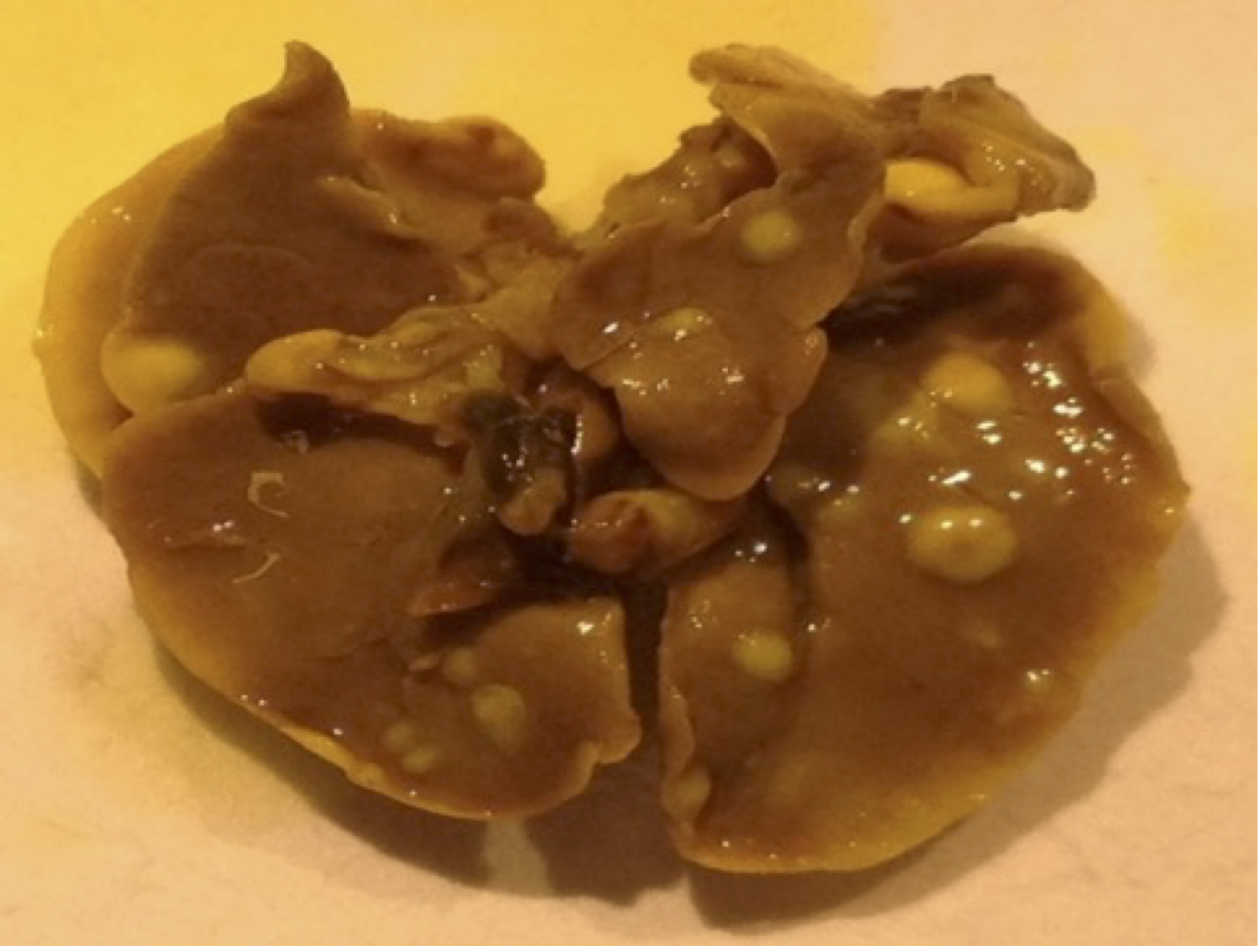
Metastases. A representative picture of the metastatic lung in the MDA-MB-231 tumor model 54 days post injection is shown.

**Fig 5:**
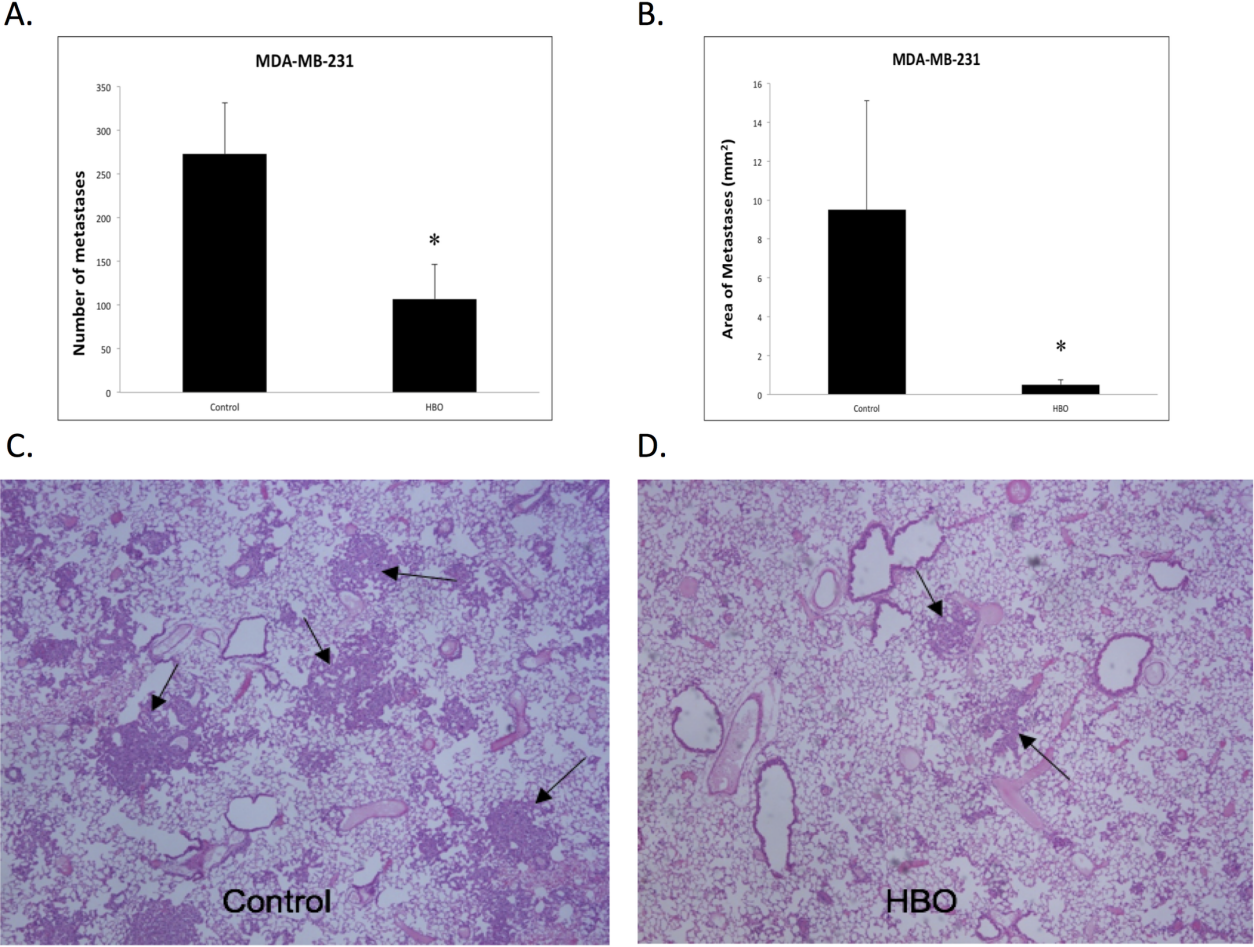
Lung metastases after hyperbaric oxygen treatment. The effect of hyperbaric oxygen treatment on the metastatic development of triple negative MBA-MB-231 breast cancer cells in vivo after 54 days. 5 x 10^5^ cells were injected in the mammary fat pad in the groin area. Mice were treated with 2.5 bar pure oxygen, 90 min each time, every third day starting 7 day post injection over a period of 54 days. Histomorphometric quantification of metastasis number (A) and area (B) in H&E stained lung sections of HBOT and controls is shown. Mean ± SD. *p<0.02. A representative lung section from one control (C) and HBOT (D) is shown.

Few studies concerning the relationship between HBOT and metastasis exists due to an old perception that HBOT would potentiate metastasis via enhanced angiogenesis. Thus, to evaluate whether HBOT has an effect on the metastatic potential of triple negative MDA-MB-231 tumors, H & E stained organ sections were evaluated. Macroscopic surface metastases were observed in all the lungs from both HBOT and control mice (Fig 4). However, there was a significant difference in the ability of primary tumor cells to metastasize to the lungs between the HBOT and controls (Fig 5). The number, as well as the area of the metastatic lesions were significantly reduced in the HBOT group (Fig 5, panels A-B). No metastatic lesions in liver nor bone were detected in either group at the time the experiements were terminated (54 days post injection).

### The effect of HBOT on Epithelial to Mesenchymal Transition (EMT) signalling

**Fig 6:**
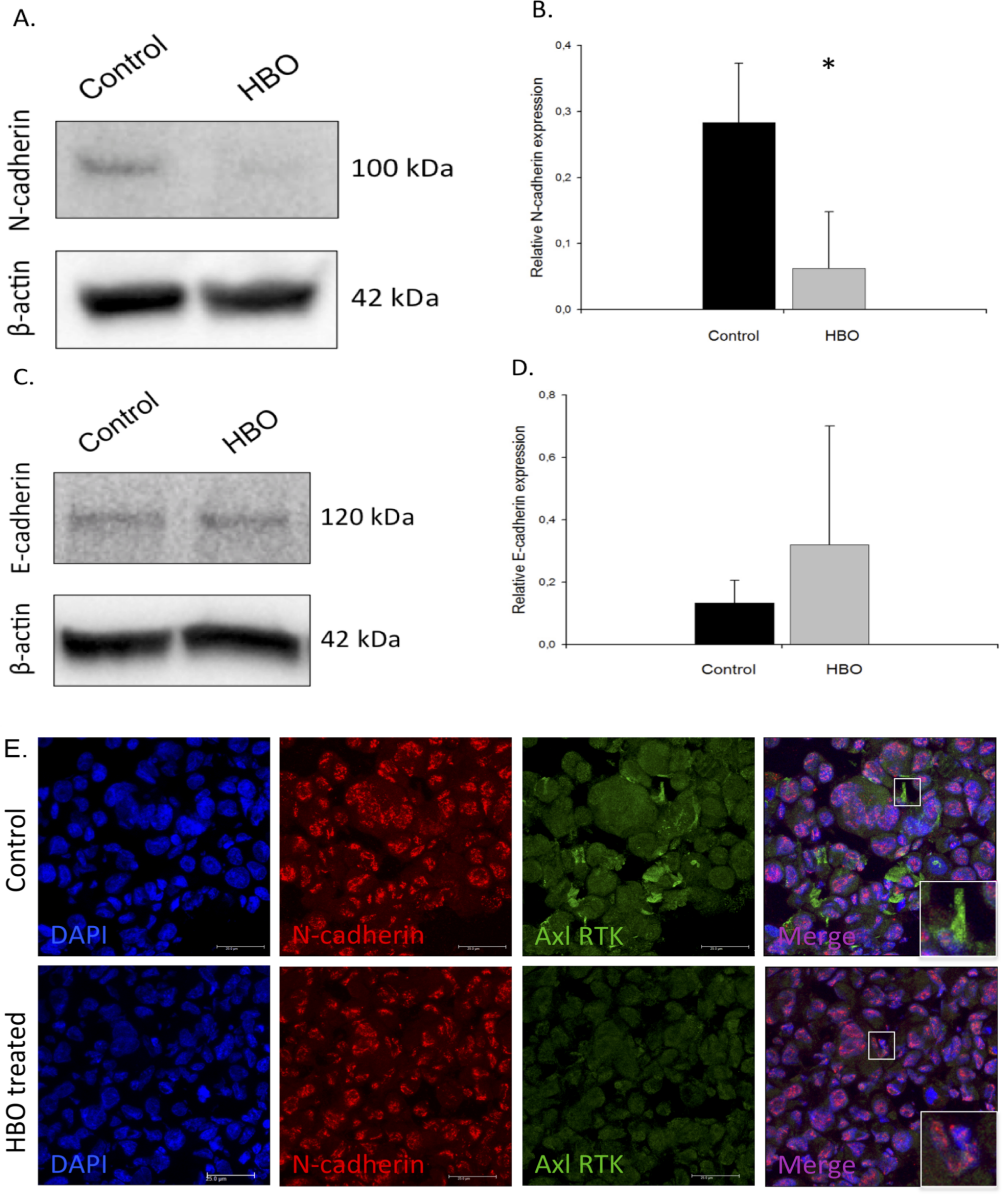
Epithelial to mesenchymal transition after hyperbaric oxygen treatment. MDA-MB-231 tumors constitutively express E-cadherin (A) and N-cadherin (C) in western blot lysates in both groups. The expression of E-cadherin (B) and N-cadherin (D) when adjusted according to loading control (β-Actin) in control (n = 5 tumors) and HBOT (n = 5 tumors) is also shown. Mean ± SD. *p>0.05. Immunofluorescent staining of cryosections from HBOT and control MDA-MB-231 tumors confirm the reduced nuclear N-cadherin and also reveals a reduction in the expression of receptor tyrosine kinase Axl (E). Scalebar: 25μm.

EMT has been indicated to play an important role in the development of cancer and metastasis and thus the specific markers E-Cadherin and N-Cadherin were investigated using western blot. Immunoblots from MDA-MB-231 tumor lysates indicated a downregulated N-cadherin expression in HBOT tumors. The downregulated N-cadherin in HBOT tumors was confirmed by volume density data, and statistical analysis showed a significant difference (p<0.02) as indicated in Fig 5. Although there was a tendency of enhanced E-cadherin expression, there were no statistically significant expression differences between the two groups (Fig 6). Immunofluorescence of cryosections from HBOT and control tumors verified the downregulation of N-cadherin in the HBOT tumors, and further revealed that N-cadherin was mainly localized to the nucleus in the MDA-MB-231 tumors. Finally, the expression of Axl was significantly downregulated (Fig 6).

### Effects of HBOT on collagen content and proportion of stroma versus tumor cells

**Fig 7:**
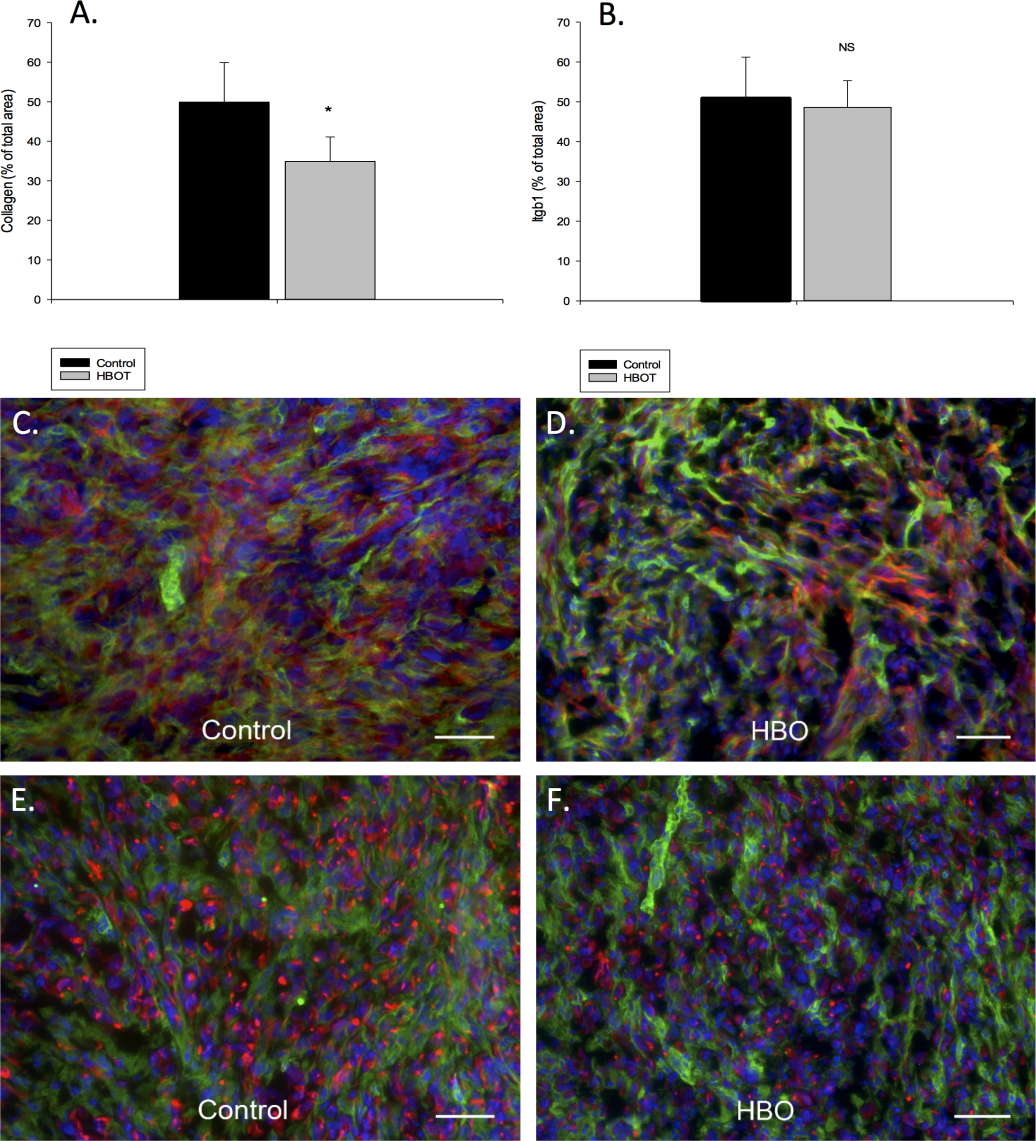
Collagen type I. The total amount of collagen type I quantified as percent of total area in both control (n = 5 tumors) and HBOT (n = 5 tumors) primary tumors (A) is shown. The total amount of ITGB1 as percent of total area in both control (n = 5 tumors) and HBOT (n = 5 tumors) primary tumors (B). A representative immunofluorescence (IF) staining picture from a control (C) and HBOT (D) tumor is shown. Green represents stroma cells (stained with antibody to mouse integrin-β1) red represents collagen type I secreted by the stromal cells (stained with antibody to mouse collagen type I), and blue shows the nuclei stained with DAPI. Scale bar represents 100 μm. Representative IF pictures are also shown to illustrate the compostion/proportion of the tumor cells (red, stained with cytokeratin 7 antibody) and the stromal cells (green, stained with integrin β1 antibody) from the control (E) and and HBOT (F) tumors, respectively. Scale bar represents 100 μm.

Analysis of immunoflouresent stained collagen type I fibrils in primary tumors, using Image J, revealed significant (p<0.001) reduced density in HBOT compared to controls as shown in Fig 7, panels A, C-D. Analysis of immunoflouresent stained Itgb1 did not show any differenes between controls and HBOT (B), neither did FSP1 as shown in S1 Fig. The amount of stroma versus tumor cells are also demonstrated in Fig 7, panels E-F, demonstrating significantly less cancer cells in the HBOT group compared to control.

## Discussion

The present study demonstrated a strong and similar primary tumor growth suppressing effect of HBOT (2.5 bar, 100% O2, a 90 min) in both triple negative and triple positive human breast cancer xenografts in mice during a period of 24 days post tumor cell injection, which is supported by a reduction in proliferation. There was however no change in angiogenesis, nor potentiating effect on the chemotherapeutic agent 5FU. The triple negative MDA-MB-231 model showed a significantly reduced number of metastasis, as well as a reduced area of the metastatic lesions following HBOT, with a concomitant reduction in the EMT marker N-Cadherin, Axl and collagen type I.

Intratumoral hypoxia is known to have negative implications for breast cancer development and is associated with a more aggressive phenotype which potentiates growth, metastasis and resistance to chemotherapy as well as radiotherapy [13, 14]. Hypoxia, via HIF-1a, causes translocation to the nucleus and increase the transcription of multiple genes associated with this aggressive behaviour. Thus, since HBOT enhanced the pO_2_ in tissues temporarily, and O_2_ is important for degrading HIF-1a, we expected reduction in tumor growth.

HBOT is commonly used for treatments including wound healing, tissue regeneration as well as after radiation injury, to enhance the pO_2_ in the otherwise hypoxic tissue [15]. The present study showed significant tumor growth suppression after HBOT in both the human MDA-MB-231 and BT-474 breast cancer model. Thus, enhanced oxygenation in the present study has been shown to induce a similar anti-cancer effect in both triple positive and triple negative breast cancers. This HBOT effect on tumor growth corresponds to what has previously been found in our laboratory in both chemically induced (DMBA) [9, 10, 12] and murine 4T1 [8, 11, 16] mammary tumors. Kluft et al. [17] showed significant inhibition of transplanted mammary carcinomas in C57 black mice after HBOT, and a strong anti-proliferative effect on different mammary cancer cells has been shown in vitro [18]. HBOT in the present study could confirm a strong anti-proliferating effect in both mammary models. Thus, HBOT seems to have a strong suppressive and/or inhibitory effect on mammary tumors in general.

HIF-1a is a key oxygen-regulator of VEGF gene expression which is important for angiogenesis and thus tumor growth [19]. Differences in tumor blood vessel density is therefore one possible cause for the differences in size between the controls and HBOT measured in MDA-MB-231 and BT-474 tumors. Although an anti-angiogenesis effect of HBOT has previously been found in other breast cancer models [8, 9] and gliomas in vivo [20], the blood vessel density and diameter was unchanged in the two human breast cancer models investigated in the present study. We therefore concluded that the anti-tumor effect found, in these two human breast tumor models after HBOT is not due to anti-angiogenesis. It could potentially be due to reduced proliferation of tumor cells, as shown in DMBA mammary tumors *in vivo* [9, 12] and *in vitro* in different mammary tumor cells [18] after HBOT. This is also indicated by the proportion of stroma/tumor between the two groups in the present study and it could also be due to enhanced apoptosis as shown previously after HBOT [9, 12].

### No potentiating effect on 5FU

HBOT is used as an adjuvant treatment for various diseases and pathological conditions and it is shown to overcome hypoxia in tumors [21]. HBOT has previously been demonstrated to enhance the effect of chemotherapeutics in different solid tumors [10, 12, 22–25]. Thus, since conventional chemotherapy is the mainstay adjuvant systemic treatment for early triple negative patient, we investigated whether it it could be potentiated by HBOT. However, the combined HBOT and 5FU therapy caused approximately the same degree of tumor growth inhibition as HBOT alone in the present MDA-MB-231 model. Thus, HBOT did not potentiate the uptake nor enhance the effect of chemotherapy in the triple-positive MDA-MB-321 tumor model. This indicates that HBOT influences the effect of 5FU differently in different tumor models, for reasons currently unknown.

### Metastasis

There are only a few studies on HBOT and metastasis likely due to an old perception that it would facilitate tumor growth and spread. The present study on the very aggressive, highly metastatic, triple-negative MDA-MB-231 tumors showed a significant reduction in both number and area of metastasis after sixteen, 90 min HBOT at 2.5 ATA. This is in accordance with a study on triple negative 4T1 murine breast cancer showing a decrease in lung colonies after HBOT (2.8 ATA) [26] and similarily in a study on rats (3 ATA) with intravenous tumor cell injection, where it was found a strong reduction in metastasis of the lung but no effect on tumor cells in the hind leg. They suggested that the response was due to a local oxygen effect in the lung [27]. Reduction in metastasis has also been found after HBOT in the lungs after i.v. injection of mammary gland-derived carcinoma cells [28] and in mice with murine LM8 osteosarcoma tumours after HBOT [29]. Based on the above, we argue that it is important to re-evaluate the effect of HBOT on metastatic potential since it seems to have a strong suppressive effect on at least lung metastasis in different animal breast cancer models.

Advanced stages of cancer is associated with extensive deposition of collagen, particular type I, in the ECM surrounding the tumor cells [30]. Collagen I is the principal collagen subtype in breast cancer and a high collagen fiber density is associated with aggressive disease and stimulation of tumor cell migration [31]. One reason for the reduced number and area of metastases after HBOT could therefore be the significant reduction in collagen I found after HBOT in the present study. Since there was no difference in Itgb1 nor in fibroblasts using FSP1 between control and HBOT we belive that the lower collagen production in HBOT tumor samples may be due to the ratio change of the collagen-producing stromal cell, like CAFs, rather than the change in total number of stromal cells. Hypoxia has been shown to be involved in Epithelial-Mesenchymal Transition (EMT) in different cancers in vivo [32, 33]. EMT is identified by epithelial cells changing their originally stable cobble-stone morphological appearance, their cell-cell adhesion properties and lack of motility to a more elongated shape with motile, invasive features [34]. Tumor progression and invasion are associated with this enhanced capacity to promote extracellular matrix remodelling and thus migration [35]. Cells with mesenchymal properties can be identified by the loss of epithelial markers such as E-cadherin, and increased expression of key mesenchymal markers, such as N-Cadherin, Snail, Twist and Vimentin [36, 37]. Previously we have shown HBOT to induce total mesenchymal to epithelial transition (MET) in DMBA tumors in rats [12] indicating a shift towards a less aggressive phenotype. In the present MDA-MB-231 model, we found a significant reduction in N-cadherin after HBOT. In most types of cancer, high N-cadherin expression correlates with cell motility, invation and metastasis [38]. Furthermore, N-cadherin was demonstrated to be localized to the nucleus in our experimental tumor model, and aberrant N-cadherin in the nucleus has been shown to be necessary for cell migration during EMT [39]. And although nuclear localisation of N-cadherin has rarely been described in human solid tumors, it has been shown to correlate with poor prognosis in nasopharyngeal carcinoma (NPC) [40]. In this study, a high expression of nuclear N-cadherin predicted a poorer survival in patients with late stage disease, and multivariate analysis showed nuclear N-cadherin to be an independent prognostic marker for NPC patients. We also demonstrated a reduction in the expression of Axl RTK in the HBOT tumors. Axl RTK has been correlated with poor outcome and drug resistance in a wide range of cancer types, and has been shown to be necessary for maintaining tumor EMT [41]. Axl RTK has been shown to be upregulated in human breast cancer samples, and to be associated with a reduced patient survival [42, 43]. Furthermore, Axl RTK expression remained an independent negative prognostic factor in a multivariate analysis including basic prognostic factors, and a hazard ratio of 3.27 were detected between cases with high and low Axl expression [43]. In an experimental tumor model, the authors demonstrated that Axl knockdown completely prevented the spread of highly metastatic breast carcinoma cells from the mammary gland to lymph nodes and several major organs and increased overall survival. Taken together, it is likely to assume based on the findings from our study that enhanced oxygen could be critically involved in MET transition of developing breast tumors, and also be implicated in the reduced metastatic potential observed after HBOT in the present study.

## Conclusion

Despite the different behavior and prognosis of the triple positive and triple negative subtypes of breast cancer, the HBOT had a strong and similar primary tumor growth suppressive effect on both tumor types, indicating they share a common oxygen dependent anti-tumor mechanism. Furthermore, the triple negative model showed significantly reduced number and area of metastases after HBOT with a reduction in the EMT markers, N-cadherin, Axl and collagen type I. Thus, as hypoxia is a negative prognostic factor for breast cancer patients, hyperoxia may provide a favourable adjuvant therapeutic approach for breast cancer growth and progression which should be evaluated further.

## Supporting information

S1 Fig Amount of fibroblast-specific protein 1. (TIF)

S1 Checklist NC3Rs ARRIVE guidelines checklist. (PDF)
