# Supplementary figures and images for: Oxygen-dependent regulation of tumor growth and metastasis in human breast cancer xenografts
Source: PLoS One. 2017 Aug 23;12(8):e0183254. doi: 10.1371/journal.pone.0183254 (PMC5568407; doi:10.1371/journal.pone.0183254)

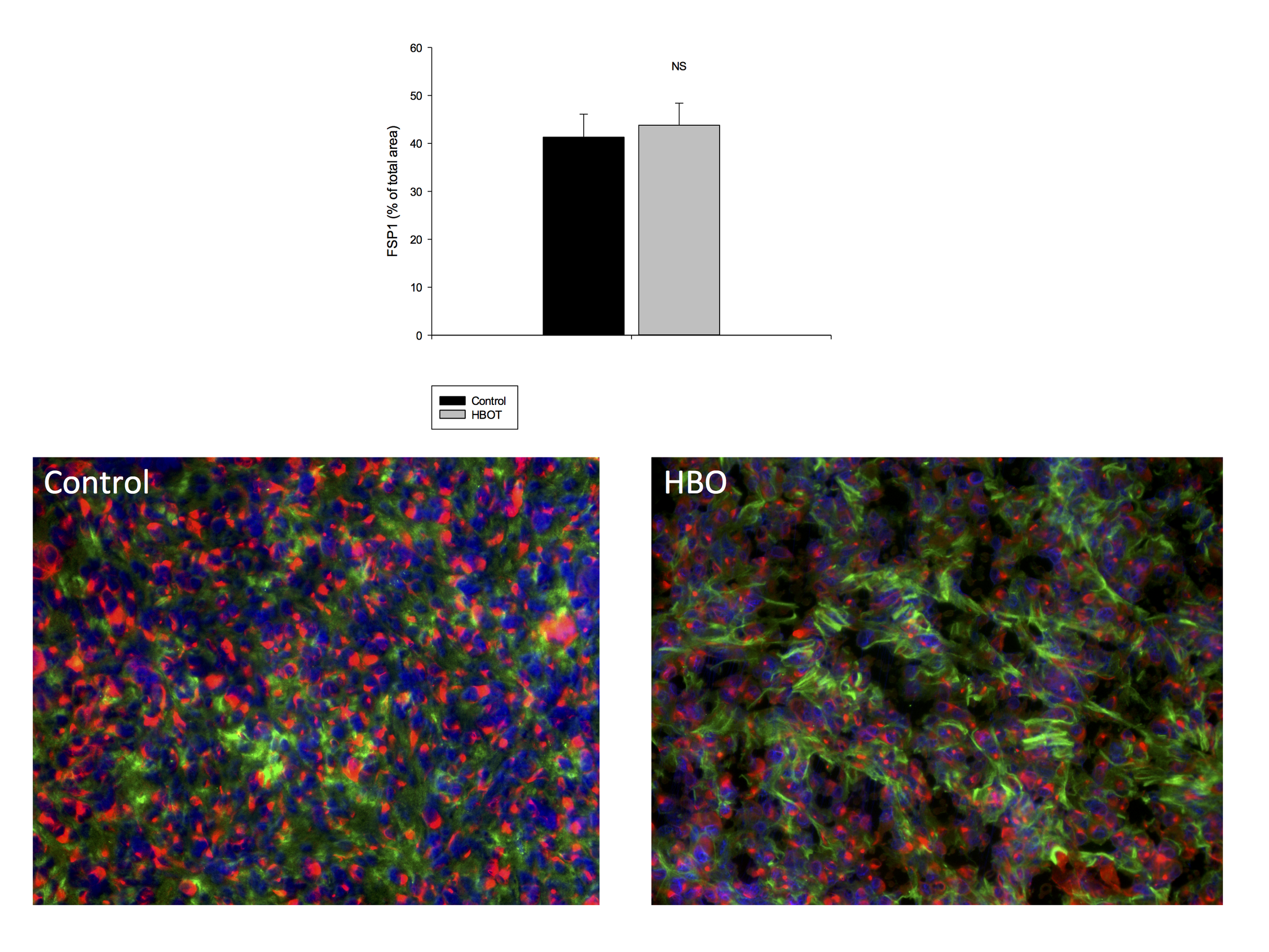

Supplement: S1 Fig — (TIF) [file pone.0183254.s001.tif]
